# The effects of phosphanegold(I) thiolates on the biological properties of *Acanthamoeba castellanii* belonging to the T4 genotype

**DOI:** 10.1186/s12952-017-0070-7

**Published:** 2017-04-03

**Authors:** Ruqaiyyah Siddiqui, Farhat Abjani, Chien Ing Yeo, Edward R. T. Tiekink, Naveed Ahmed Khan

**Affiliations:** 1 Department of Biological Sciences, Faculty of Science and Technology, Sunway University, 47500 Bandar Sunway, Selangor, Malaysia; 2 Research Centre for Crystalline Materials, Sunway University, 47500 Bandar Sunway, Selangor, Malaysia

**Keywords:** *Acanthamoeba*, Gold compounds, Cytotoxicity assays, Zymography, Encystation, Excystation

## Abstract

**Background:**

Gold compounds have shown promise in the treatment of non-communicable diseases such as rheumatoid arthritis and cancer, and are considered of value as anti-microbial agents against Gram-negative and Gram-positive bacteria, and have anti-parasitic properties against *Schistosoma mansoni*, *Trypanosoma brucei*, *Plasmodium falciparum*, *Leishmania infantinum*, *Giardia lamblia*, and *Entamoeba histolytica*. They are known to affect enzymatic activities that are required for the cellular respiration processes.

**Methods:**

Anti-amoebic effects of phosphanegold(I) thiolates were tested against clinical isolate of *A. castellanii* belonging to the T4 genotype by employing viability assays, growth inhibition assays, encystation assays, excystation assays, and zymographic assays.

**Results:**

The treatment of *A. castellanii* with the phosphanegold(I) thiolates tested (i) had no effect on the viability of *A. castellanii* as determined by Trypan blue exclusion test, (ii) did not affect amoebae growth using PYG growth medium, (iii) did not inhibit cellular differentiation, and (iv) had no effect on the extracellular proteolytic activities of *A. castellanii*.

**Conclusion:**

Being free-living amoeba, *A. castellanii* is a versatile respirator and possesses respiratory mechanisms that adapt to various aerobic and anaerobic environments to avoid toxic threats and adverse conditions. For the first time, our findings showed that *A. castellanii* exhibits resistance to the toxic effects of gold compounds and could prove to be an attractive model to study mechanisms of metal resistance in eukaryotic cells.

## Background

*Acanthamoeba* is a free living pathogenic protist that can cause cutaneous lesions, a vision-threatening keratitis, and a rare but fatal infection of the brain, identified as granulomatous amoebic encephalitis [1–4]. *Acanthamoeba* keratitis infection is of explicit concern given the rise in the number of wearers of contact lenses worldwide, a population susceptible to this infection. Treatment involves hourly topical application of a mixture of drugs comprising of polyhexamethylene biguanide or chlorhexidine digluconate together with propamidine isethionate or hexamidine. Moreover, chloramphenicol or neomycin is also given to prevent mixed bacterial infection [5]. Treatment lasts for several months [5, 6]. Furthermore, the treatment is problematic and cumbersome, in part due to the ability of this facultative parasite to go through phenotypic interchanging into a double-walled cyst form, which is impervious to many anti-microbial drugs and harsh conditions, and an active vegetative trophozoite stage that is more vulnerable to anti-microbials, often leading to recurrence of infection [7–9]. Consequently, there is a crucial need to develop anti-microbials targeting both the cyst stage and the trophozoite stage of *Acanthamoeba*.

**Fig. 1:**
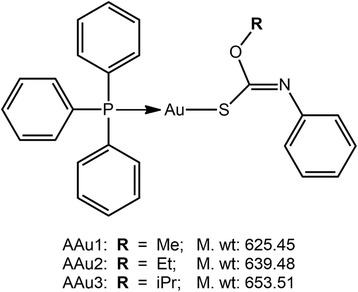
Chemical diagrams, abbreviations and molecular weights for AAu1–AAu3

Gold compounds have been well recognised for their putative properties and potential medical applications [10, 11]. For example, the assessment of the potential anti-cancer activity and the determination of signalling pathways for apoptosis of phosphane gold(I) carbonimidothioates, Ph_3_PAu[SC(OR) = NPh], R = Me, Et and iPr, and related species have been carried out recently [12–14], see Fig. 1 for chemical structures. Moreover, closely related compounds have shown potential as anti-microbial agents against Gram-positive bacteria [15]. Gold(I) compounds have potential medical applications and shown to possess anti-tumour activities [16, 17], anti-parasitic [18] and anti-microbial activities [19–21] via a variety of mechanisms including respiration. In this study, for the first time, we determined the effects of phosphanegold(I) thiolates, AAu1–AAu3, Fig. 1, on a keratitis-causing isolate of *A. castellanii* belonging to the T4 genotype. Furthermore, the effects on viability, growth, encystation and excystation are examined.

## Methods

### Chemicals

All chemicals were purchased from Sigma Labs (Poole, Dorset, England), unless otherwise stated. The phosphanegold(I) thiolates, AAu1–AAu3, were prepared and characterised using methodology as previously described [14]. The molecular structures and weights of AAu1–AAu3 are given in Fig. 1. A stock solution (10 mM) was prepared and stored at −20 °C until used. Control cultures contained the same volume of respective solvents.

### Cultures of A. castellanii

*A. castellanii* belonging to the T4 genotype (ATCC 50492) is a clinical isolate that was initially isolated from a keratitis patient and grown in 75 cm^2^ tissue culture flasks in 10 mL at a cell density of 5×10^5^ cells per mL in PYG medium [proteose peptone 0.75% (w/v), yeast extract 0.75% (w/v) and glucose 1.5% (w/v)] without shaking at 30 °C as described previously [22, 23]. At this cell density, parasites reach confluency within 48 h. Active trophozoites are attached to the bottom of the flasks while any dormant cells are non-adherent in the supernatant. To obtain trophozoites, supernatant was aspirated and 10 mL of RPMI-1640 was added. Next, flasks were placed on ice for 20 min to detach bound amoebae followed by gentle tapping and observed under the inverted microscope to ensure amoebae detachment had occurred. Finally, the parasites were collected in 50 mL tubes, followed by centrifugation at 1500×*g* for 5 min, resuspended in one mL of RPMI-1640 and used in experiments.

### Amoebicidal assays

To determine amoebicidal activity of AAu1–AAu3, *A. castellanii* trophozoites (5 × 10^5^ amoebae/0.5 mL/well) were incubated in RPMI-1640 with various concentrations of AAu1–AAu3 in 24-well plates as described previously [20–24]. Plates were incubated at 37 °C for 24 h. Following this incubation, amoebae viability was determined by adding 0.1% Trypan blue and number of live (non-stained) and dead (stained) *A. castellanii* were enumerated using a haemocytometer. The counts from *A. castellanii* incubated with RPMI-1640 alone, and the solvent alone (chloroform) were used as controls. Data are represented as the mean ± standard error of at least three independent experiments. To determine whether the effects of AAu1–AAu3 are irreversible, *A. castellanii*, 5 × 10^5^ trophozoites, were incubated with AAu1–AAu3 for 24 h as described above. After this incubation, amoebae were centrifuged for 10 min at 1,000xg and supernatant was aspirated, followed by the addition of 0.5 mL of RPMI-1640. This process was repeated 3X to remove extracellular AAu1–AAu3. Finally, *A. castellanii* were re-suspended in PYG as a food source and inoculated in 24-well plates. Plates were incubated at 37 °C for up to 72 h and re-emergence of trophozoites was considered as viable amoebae, and absence of trophozoites was considered as non-viable amoebae. In some experiments, plates were incubated for up to a week to observe the emergence of viable trophozoites.

### Amoebistatic assays

To determine the effects of AAu1–AAu3 on the growth of *A. castellanii*, assays were performed by exposing 5 × 10^5^ trophozoites to different concentrations of AAu1–AAu3 in growth medium, i.e., PYG in 24-well plates. Next, the plates were incubated at 30 °C for 48 h. For controls, 5 × 10^5^ trophozoites were inoculated in 100% PYG medium, 100% non-nutritive PBS and respective amounts of solvents plus PYG medium and incubated in the above-mentioned conditions. After this incubation, the number of amoebae was determined by haemocytometer counting. All experiments were performed at least three times in duplicate.

### Preparation of A. castellanii cysts and excystation assays

To prepare *A. castellanii* cysts, encystation was induced by inoculating 5 × 10^6^ *A. castellanii* trophozoites onto non-nutrient agar plates [prepared using 3% (w/v) bacteriological agar] and incubating at 30 °C for up to 14 days [25]. Food deprivation resulted in trophozoite transformation into the cyst form. Next, 10 mL of PBS was added to each plate. Cysts were then gently scraped off the agar surface using a cell scraper. PBS containing cysts was collected in 15 mL tube and centrifuged at 3000 × *g* for 10 min to pellet cysts. The supernatant was aspirated and cysts resuspended in RPMI-1640, enumerated using a haemocytometer and used in experiments. To determine the effects of AAu1–AAu3 on excystation, assays were performed by inoculating *A. castellanii* cysts (5 × 10^4^ cysts per mL PYG per well of 24-well plates) in the presence or absence of different concentrations of AAu1–AAu3. Plates were incubated at 30 °C and observed every 24 h under the inverted microscope for the emergence of viable trophozoites for up to 72 h.

### Encystation assays

Encystation assays were performed as described previously [25]. Briefly, 2 × 10^6^ amoebae were incubated in 0.5 mL of PBS containing 50 mM MgCl_2_ and 10% glucose (i.e., encystation trigger) per well of 24-well plates. The plates were incubated at 30 °C for 72 h without shaking. After this incubation, amoebae viability was quantified using a haemocytometer via Trypan blue exclusion assay. Next, SDS (0.5% final conc.) was added for 10 min. At this concentration, SDS solubilizes amoebae trophozoites but not cysts. Finally cysts were enumerated using a haemocytometer and used in experiments. To determine the effects of AAu1–AAu3 on encystation, assays were performed in the presence of different concentrations of drugs. Briefly 2 × 10^6^ amoebae were incubated in PBS with various concentrations of drugs and incubated at room temperature for 20 min. Following this, 50 mM MgCl_2_ and 10% glucose was added as a trigger for encystation and plates were incubated at 30 °C for 72 h. Finally, parasites counts were determined using a haemocytometer. Amoebae incubated without inhibitors and encystation trigger were used as controls. The respective amounts of solvents were used as solvent controls.

### Zymographic assays

The extracellular proteolytic activities of *Acanthamoeba* were determined using zymographic assays as previously described [26]. Briefly, *A. castellanii* were incubated in the presence or absence of various concentrations of AAu1–AAu3 for 24 h. Next day, cell-free supernatants (CM, conditioned medium) were collected by centrifugation. The CM were electrophoresed on sodium dodecyl sulfate-polyacrylamide gel electrophoresis (SDS-PAGE) containing gelatin (2 mg/mL) as a protease substrate as previously described [26]. Following electrophoresis, gels were washed in 2.5% Triton X-100 (w/v) for 60 min, then incubated in developing buffer (50 mM Tris–HCl, pH 7.5, containing 10 mM CaCl_2_) at 37 °C overnight. Next day, gels were stained with Coomassie Brilliant Blue. Areas of gelatin digestion were visualised as non-staining regions in the gel.

### Statistical analysis

Statistical significance for differences was evaluated using 2 sample *t*-test; two-tailed distribution, comparing the mean of two independent groups in Excel. A critical value of P < 0.05 was used for all analysis. For graphical representation of the data, y-axis error basis indicate the standard error of the data for each point on the figure.

## Results

### Phosphanegold(I) thiolates, AAu1–AAu3, did not affect A. castellanii trophozoites viability

**Fig. 2:**
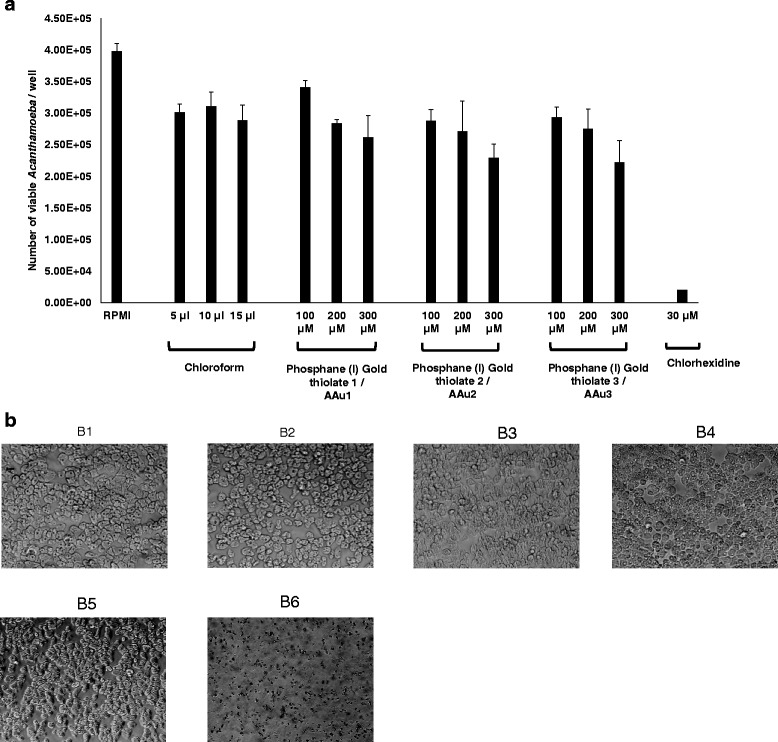
**a** The effects of AAu1–AAu3 on the viability of *A. castellanii* belonging to T4 genotype. Briefly *A. castellanii* (5 × 10^5^ trophozoites) were incubated with gold thiolates at 37 °C for 24 h. Next day, Trypan blue exclusion assays were performed and amoebae were counted using haemocytometer. Note that none of the compounds shows significant effect on the viability of *A. castellanii* as compared to control. The results represent the mean ± standard error of three different experiments performed in duplicates. **b** Representative effects of AAu1–AAu3 on survival of *A. castellanii*. Briefly, *A. castellanii* (5 × 10^5^ trophozoites) were incubated with AAu1–AAu3 at 37 °C for 24 h and were counted using a heamocytometer. Next, drugs-treated amoeba were washed and re-inoculated in fresh PYG at 37 °C for up to 24 h and observed under a microscope. The results are representative of three independent experiments. B1 is Amoeba alone; B2 is solvent alone (chloroform 15 μL); B3 is AAu1 (300 μM); B4 is AAu2 (300 μM); B5 is AAu3 (300 μM); B6 is chlorhexidine (300 μM)

To ascertain the effects of AAu1–AAu3, amoebicidal assays were performed as stated in Materials and Methods. The results revealed that AAu1–AAu3 did not exhibit anti-amoebic effects against *A. castellanii* trophozoites (Fig. 2a and b). In the presence of 100, 200 and 300 μM AAu1, the number of viable amoebae was 3.41 × 10^5^ ± 1.12 × 10^4^, 2.84 × 10^5^ ± 5.51 × 10^3^ and 2.62 × 10^5^ ± 3.47 × 10^4^, respectively. However, this was not significant when compared to the respective solvent controls (5, 10 and 15 μL chloroform). Likewise, for 100, 200 and 300 μM AAu2, the number of viable amoebae was 2.88 × 10^5^ ± 1.75 × 10^4^, 2.72 × 10^5^ ± 4.73 × 10^4^ and 2.30 × 10^5^ ± 2.14 × 10^4^, respectively. For 100, 200 and 300 μM AAu3, the number of viable amoebae was 2.94 × 10^5^ ± 1.56 × 10^4^, 2.76 × 10^5^ ± 3.09 × 10^4^ and 2.23 × 10^5^ ± 3.39 × 10^4^, respectively (Fig. 2a). Overall, the results showed no effects of AAu1–AAu3 on amoebae viability.

### Phosphanegold(I) thiolates, AAu1–AAu3, did not exhibit amoebistatic effects against A. castellanii trophozoites

**Fig. 3:**
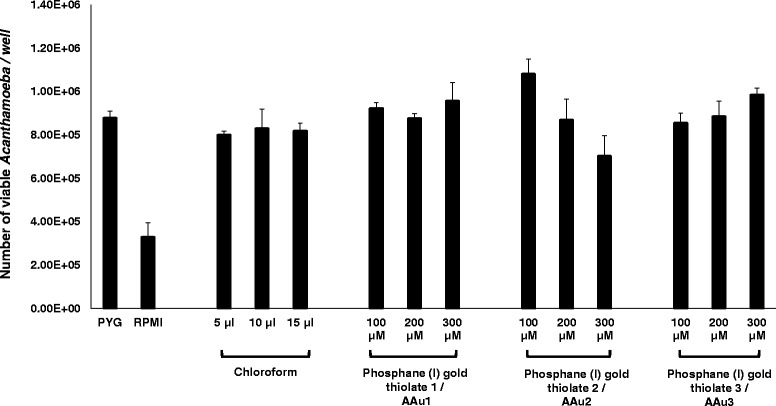
The effects of AAu1–AAu3 on the growth of *A. castellanii* belonging to T4 genotype. Briefly *A. castellanii* (5 × 10^5^ trophozoites) were incubated with AAu1–AAu3 in growth medium, PYG at 37 °C for 24 h. After this period, amoebae were counted using haemocytometer. Note that none of the trial compounds shows significant effect on the growth of *A. castellanii* as compared to control. The results represent the mean ± standard error of three different experiments performed in duplicates

Amoebistatic assays were performed in the presence or absence of AAu1–AAu3. When incubated in 100% growth medium, the number of amoebae increased from 5 × 10^5^ to 8.78 × 10^5^ ± 3.21 × 10^4^ (Fig. 3). In contrast, amoebae incubated in non-nutritive RPMI medium had no growth stimulatory effect but exhibited reduced number of amoebae i.e., the amoebae count decreased from 5 × 10^5^ to 3.29 × 10^5^ ± 6.63 × 10^4^ (Fig. 3). For AAu1–AAu3, the results revealed that there were no amoebistatic effects against *A. castellanii* even at 300 μM concentrations. For AAu1–AAu3, the number of amoebae increased from 5 × 10^5^ to 9.56 × 10^5^ ± 8.42 × 10^4^, 7.02 × 10^5^ ± 9.38 × 10^4^ and 9.85 × 10^5^ ± 3.07 × 10^4^, respectively at 300 μM.

### Phosphanegold(I) thiolates, AAu1–AAu3, did not affect excystation in A. castellanii

**Fig. 4:**
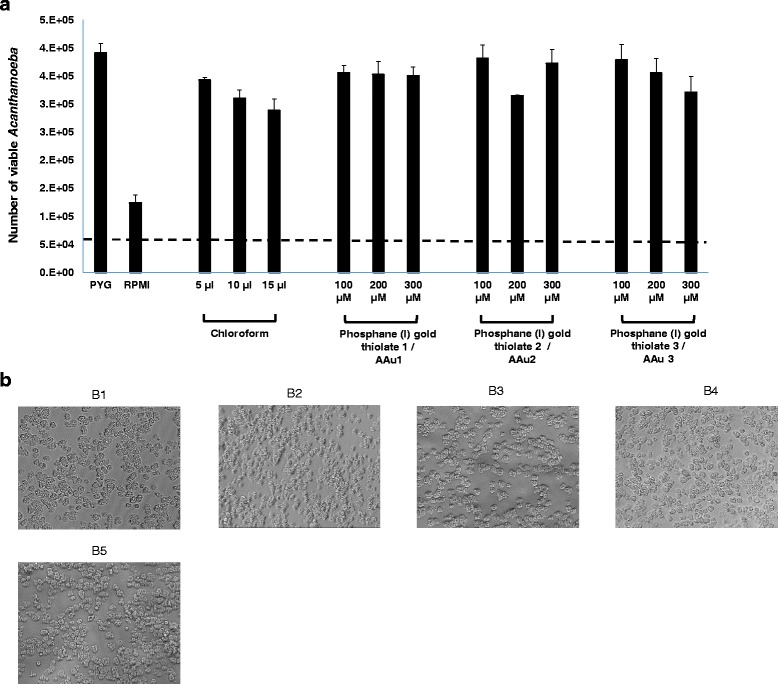
**a** The effects of AAu1–AAu3 on excystation of *A. castellanii* belonging to T4 genotype. Briefly *A. castellanii* (5 × 10^4^ cyst) were incubated with AAu1–AAu3 in growth medium, PYG at 37 °C for 48 h. After this period, amoebae were counted using a haeamocytometer. Note that AAu1–AAu3 were unable to inhibit excystation. The dotted line represents the original inoculum. The results represent the mean ± standard error of two different experiments performed in duplicates. **b** Representative effects of AAu1–AAu3 on excystation of *A. castellanii*. The results are representative of three independent experiments. B1 is Amoeba alone; B2 is solvent alone (chloroform 15 μL); B3 is AAu1 (300 μM); B4 is AAu2 (300 μM); B5 is AAu3 300 (μM)

When incubated in growth medium, the number of amoebae increased from 5 × 10^4^ to 3.91 × 10^5^ ± 1.63 × 10^4^ as compared to 5 × 10^4^ to 1.24 × 10^5^ ± 1.38 × 10^4^ in RPMI medium, which is a non-nutritive medium (Fig. 4a). However, for AAu1–AAu3, the number of amoebae increased from 5 × 10^4^ to 3.50 × 10^5^ ± 1.63 × 10^4^, 3.73 × 10^5^ ± 2.50 × 10^4^ and 3.21 × 10^5^ ± 2.81 × 10^4^, respectively at 300 μM (Fig. 4a). Nonetheless, this was not significant when compared to the respective growth medium control and the results revealed that none of the compounds tested had any effects on excystation, and amoebae were able to excyst at rates comparable to controls (Fig. 4b)*.*

### Phosphanegold(I) thiolates, AAu1–AAu3, did not affect encystation in A. castellanii

**Fig. 5:**
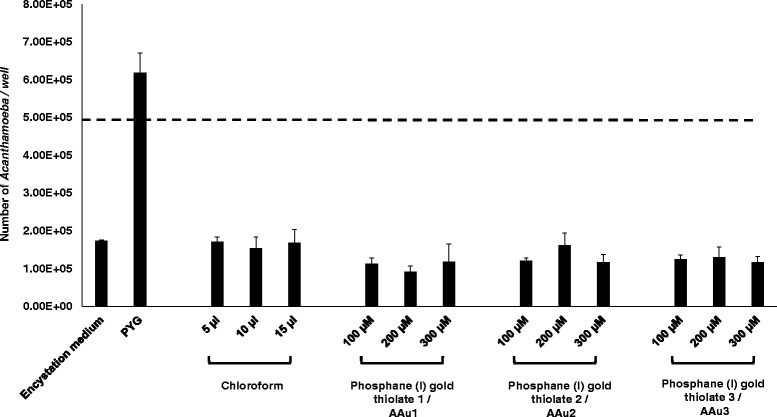
The effects of AAu1–AAu3 on encystation of *A. castellanii* belonging to T4 genotype. Briefly, 5 × 10^5^ trophozoites were incubated with AAu1–AAu3 in encystation medium (50 mM MgCl_2_ and 10% glucose) as described in “Methods”. The dotted line represents the original inoculum. The results are expressed as the mean ± standard error of three independent experiments performed in duplicate

To determine the effects of AAu1–AAu3 on *A. castellanii* encystation*,* assays were performed in the presence and absence of these compounds. When incubated in encystation medium, the number of amoebae decreased from 5 × 10^5^ to 1.73 × 10^5^ ± 2.50 × 10^3^ (Fig. 5). However, for AAu1–AAu3, the number of amoebae was reduced from 5 × 10^5^ to 1.18 × 10^5^ ± 4.75 × 10^4^, 1.17 × 10^5^ ± 2.06 × 10^4^ and 1.17 × 10^5^ ± 1.44 × 10^4^, respectively, at 300 μM (Fig. 5). However, this was not significant when compared to the respective encystation medium control. The results revealed that none of the trial compounds tested had any effects on encystation.

### Phosphanegold(I) thiolates, AAu1–AAu3, did not effect A. castellanii extracellular proteolytic activity

**Fig. 6:**
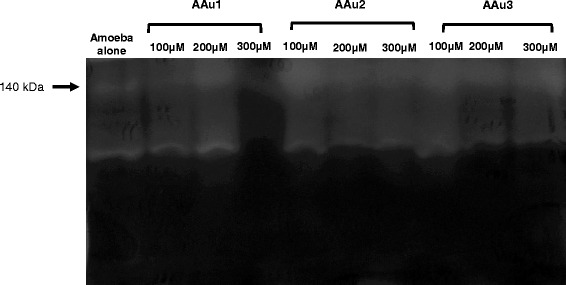
The effects of AAu1–AAu3 on extracellular proteolytic activity of *A. castellanii* belonging to T4 genotype. Zymographic assays were performed using gelatin as a substrate to determine the effects of AAu1–AAu3 on extracellular proteases of *A. castellanii* using 100, 200 and 300 μM concentrations. The results revealed that none of AAu1–AAu3 inhibited *A. castellanii* proteases when compared with amoeba in RPMI alone. The results are representative of three independent experiments

To determine the effect of AAu1–AAu3 on the extracellular proteases of *A. castellanii*, zymographic assays were performed using gelatin as substrate as described in materials and methods. In the absence of any trial compound*, A. castellanii* exhibited proteolytic activities and a visible band of 140 kDa was observed (Fig. 6). Similarly, both, *A. castellanii* treated in the presence of different concentrations of AAu1–AAu3 and in RPMI alone exhibited extracellular proteases at similar levels (Fig. 6).

## Discussion

Gold(I) complexes have potential medical applications [10, 11]. Thus, gold(I) derivatives have been explored for anti-tumour activity [16, 17] as well as anti-parasitic [18] and anti-microbial agents [19–21]. Gold has properties such as high thermal/chemical stability and resistant to oxidation, yet is mechanically soft with high electric conductivity enabling its applications in several disciplines ranging from healthcare to engineering. For example, gold compounds have been successfully used in the treatment of rheumatoid arthritis and are shown to slow down the progression of rheumatic disorder [27, 28]. Many of the biologically active gold(I) compounds contain thiolates and/or phosphane as ligands [10, 11, 16, 17, 21] and inhibit thioredoxin reductase [29, 30]. More recently, it is shown that the gold(I) compounds exhibit anti-parasitic activities such as targeting *Schistosoma mansoni* [31], *Trypanosoma brucei* [32], *Echinococcus granulosus* [33], *Plasmodium falciparum* [34], *Leishmania infantinum* [35] *Giardia lamblia* [36], and *Entamoeba histolytica* [37]. Furthermore, it was shown that gold(I) compounds target *E. histolytica* by inhibiting thioredoxin reductase activity [37]. The anti-bacterial activities of gold(I) compounds showed that these compounds affect *Clostridium difficile* and *Treponema denticola* by disrupting the selenium metabolism by targeting selenoproteins required for energy [38, 39], while *Staphylococcus aureus* growth is inhibited by gold(I) compounds [40]. Other studies proposed targets including the inhibition of mitochondrial enzymes and of the proteasome compounds [41, 42] and the inhibition of the zinc finger protein poly (adenosine diphosphate (ADP) ribose) polymerase 1 (PARP-1) [43, 44]. Notably, PARP’s are crucial proteins that are important in drug resistance in cancer as they play an essential role in DNA repair by detecting DNA strand breaks and catalyzing poly (ADP-ribosylation) [45]. Other biological targets of gold(I) compounds with prokaryotic and eukaryotic cells are yet to be discovered.

Based on these findings, it was logical to test the anti-amoebic effects of phosphanegold(I) thiolates, AAu1–AAu3, on the biological properties of *A. castellanii* belonging to the T4 genotype. The results revealed that AAu1–AAu3 did not show any effects on the biological properties of the parasite. This was determined by performing (i) viability assays using Trypan blue exclusion test, (ii) amoebae growth using PYG growth medium, (iii) cellular differentiation using encystation and excystation assays and (iv) enzymatic activities by determining extracellular proteases profiles. The reported results are highly reproducible and consistently showed that AAu1–AAu3 do not affect the biological properties of *A. castellanii*. There could be several explanations for the findings observed in this study. For example, the mode of action of gold requires it to enter the cell, via the hydrophobic cell membrane, to produce damage, most likely through transmembrane proteins that may be different in *A. castellanii*. Notably, gold(I) compounds are well known to affect enzymatic activities that are required for the cellular respiration processes. Being one of the most ubiquitous protists, the natural habitat of *Acanthamoeba* is the environment with diverse respiratory mechanisms and wide exposure to metals, thus *Acanthamoeba* is likely to possesses mechanisms to inhibit the toxic effects exerted by metals. *A. castellanii* is well known as a versatile respirator and possesses several mitochondria per cell and respiratory mechanisms that adapt to various aerobic and anaerobic environments to dodge toxic threat and adverse conditions. It is possible that the toxic effects of metals are compensated by switching the type of respiration or the use of an efflux system to rid toxic metals. Future studies are needed to test higher concentration of phosphanegold(I) thiolates compounds and/or in combining phosphanegold(I) thiolates with current anti-amoeba drugs, such as chlorhexidine to determine their improved efficacy against pathogenic *Acanthamoeba*. Overall, these findings suggest that *Acanthamoeba* exhibits resistance to toxic effects of gold(I) compounds and could prove to be an attractive model to study mechanisms of metal resistance in eukaryotic cells.

## Conclusions

Although gold compounds have shown promise in the treatment of non-communicable diseases such as rheumatoid arthritis, anti-tumour activities, as well as antibacterial properties, and anti-parasitic properties against protozoan pathogens, *T. brucei*, *P. falciparum*, *L. infantinum*, *G. lamblia*, and *E. histolytica*, often by targeting respiration pathways, our studies demonstrated that *A. castellanii* exhibited resistance against their toxic effects. The gold derivatives tested had no effect on the viability of *A. castellanii*, did not inhibit amoebae growth, or cellular differentiation processes or extracellular proteolytic activities. As *Acanthamoeba* is a versatile respirator, it can adapt to various aerobic and anaerobic environments to avoid toxic threats. Our studies suggest that *Acanthamoeba* could prove to be a useful model to study mechanisms of metal resistance in eukaryotic cells.
